# Bone Marrow Stem Cell Derived Paracrine Factors for Regenerative Medicine: Current Perspectives and Therapeutic Potential

**DOI:** 10.1155/2011/207326

**Published:** 2010-12-06

**Authors:** Tom J. Burdon, Arghya Paul, Nicolas Noiseux, Satya Prakash, Dominique Shum-Tim

**Affiliations:** ^1^Biomedical Technology and Cell Therapy Research Laboratory, Department of Biomedical Engineering, Faculty of Medicine, Duff Medical Building, 3775 University Street, McGill University, QC, Canada H3A 2B4; ^2^Dèpartment de Chirurgie Cardiaque, Centre Hospitalier de l'Université de Montréal, Pavillon Hôtel-Dieu, Université de Montréal, Montréal, QC, Canada H3C 3J7; ^3^Divisions of Cardiac Surgery and Surgical Research, The Montreal General Hospital, McGill University Health Center, QC, Canada H3G 1A4; ^4^The Royal Victoria Hospital, Suite S8.73.B, 687 Pine Avenue West, Montréal, QC, Canada H3A 1A1

## Abstract

During the past several years, there has been intense research in the field of bone marrow-derived stem cell (BMSC) therapy to facilitate its translation into clinical setting. Although a lot has been accomplished, plenty of challenges lie ahead. Furthermore, there is a growing body of evidence showing that administration of BMSC-derived conditioned media (BMSC-CM) can recapitulate the beneficial effects observed after stem cell therapy. BMSCs produce a wide range of cytokines and chemokines that have, until now, shown extensive therapeutic potential. These paracrine mechanisms could be as diverse as stimulating receptor-mediated survival pathways, inducing stem cell homing and differentiation or regulating the anti-inflammatory effects in wounded areas. The current review reflects the rapid shift of interest from BMSC to BMSC-CM to alleviate many logistical and technical issues regarding cell therapy and evaluates its future potential as an effective regenerative therapy.

## 1. Introduction

The objective of stem cell regenerative therapy is to treat damaged organ tissues by avoiding the processes of cell death and/or inadvertent remodeled Tissue [1]. Great optimism has resulted from bone marrow derived stem cell (BMSC) research ever since it showed to contribute significantly to the reestablishment of some functionality in injured organs [2, 3]. The mechanisms by which stem cells function and reverse the effects of cell death include differentiation, cell fusion, and secretion of cytokines or paracrine effects [1, 4–6]. More specifically, studies injecting BMSCs have shown to improve functionality of ischemic tissue by promoting neovascularization, inhibition of apoptosis and anti-inflammation, better localization and homing of therapeutic cells, and stimulation of endogenous cells differentiation and proliferation [7–10]. Although a lot of research has been focused on the ability of stem cells to differentiate within the injured areas, more recent research suggests other mechanisms may be more therapeutically relevant. It will be argued that understanding paracrine mechanisms, mediated by stem cells, is essential if stem cell regenerative therapy is ever to reach clinical importance.

Indeed, understanding the therapeutic effects of regenerative therapy using BMSCs becomes more relevant when we look at the paracrine factors, which are secreted by BMSCs. For example, the frequency of stem cell engraftment and the number of newly generated cardiomyocytes or vascular cells are too insignificant to represent the remarkable cardiac functional improvement attributed to fusion or differentiation alone [11]. In addition, *in vivo* transplanted cells are exposed to local immune cells and soluble mediators, which influence the cells behavior in an unpredictable manner in the microenvironment. Thus, it is necessary to further understand the potential benefits of maximizing the paracrine effects for regenerative therapy. This review will take an in-depth look at specific mechanisms regulated by these factors and potential therapeutic applications of BMSC-CM and paracrine factors secreted by BMSCs. 

BMSCs include many populations of progenitor cells: hematopoietic stem cells (HSC), mesenchymal stem cells or stromal cells (MSC), side population cells, and multipotent adult progenitor cells [12]. BMSCs can be aspirated, and the entire mononuclear cell fraction containing a heterogeneous mix of progenitor and inflammatory cells is obtained through density-gradient centrifugation using Ficoll. MSCs, which are commonly used in the lab, are present at a concentration several folds lower than their hematopoietic counterparts, representing approximately 0.01% of the total nucleated marrow cell population. They are separated from other cells in culture by their preferential attachment to plastic surfaces [13–16]. MSCs do not express hematopoietic or endothelial cell surface markers. MSCs are expandable in culture without losing their differentiation potential and constitute an unlimited pool of transplantable cells. They are multipotent and can differentiate into multiple lineages, including fibroblasts, osteoblasts, chondroblasts, and adipocytes [17–23]. Differentiation of MSCs to cardiomyocyte-like cells has been observed *in vitro* under specific conditions and *in vivo* after injection into the myocardium [24–27]. 

## 2. Emerging Role of BMSCs for Cell and Tissue Regeneration Therapy

MSCs are particularly suitable for cell therapy because of easy isolation, high expansion potential giving unlimited pool of transplantable cells, low immunogenicity, amenability to *ex vivo* genetic modification, and multipotency [24, 28, 29]. Although MSCs undergo lineage-specific differentiation to generate bone, fat, and cartilage, they have been reported to transdifferentiate into defined ectodermal and endodermal tissues [30]. Furthermore, MSCs are available for autologous therapies, can bypass immune rejection, and are inherently migratory. Differentiation of MSCs into cells expressing cardiomyocytes markers has been obtained *in vitro* and *in vivo * [26, 27, 29, 31–36]. They are also known to secrete a variety of biologically active factors and promote collateral blood flow development through paracrine mechanisms [37–44]. Moreover, bone marrow stromal cells are capable of differentiation, regeneration of infarcted myocardium, induction of myogenesis, and promotion of angiogenesis. These cells can potentially differentiate into cardiomyocytes *in vivo* and even express functional adrenergic and muscarinic receptors [45, 46]. Moreover, conditioned medium collected from MSC (MSC-CM) promotes *in vitro* proliferation and migration of endothelial cells and vascular smooth muscle cells, and enhances blood flow recovery of ischemic hindlimb [37, 43, 44]. Following exposure to hypoxia and serum starvation, MSCs are stimulated to secrete several growth factors and cytokines [47]. Noiseux et al. have shown that injection of MSC-CM either directly into infarcted heart by intramyocardial or intraperitoneal injections improve myocardial function and repair [48–56]. The mechanism by which MSCs may exhort beneficial effects is debated but is certainly synergistic [57]. 

Although the mechanisms and the therapeutic benefits have not yet been fully elucidated, general consensus states that adult stem cells, particularly BMSCs, are very safe. This is based on their usage in human studies, which have shown that they can be safely cultured *in vivo* with little risk of malignant transformations [6]. Pitfalls to be considered when working with MSCs are that transplantation of MSCs alone can generate local immune responses and disrupt homeostasis within tissue milieu by causing the release of inflammatory mediators such as cytokines [32]. Moreover, the therapeutic ability of BMSCs to suppress immune responses may indirectly promote metastatic tumour proliferation and growth. In addition, cytogenic abnormalities, such as tumour differentiation, have been observed in MSCs which have undergone extensive culturing and long-term passaging [6]. Another area of concern involving BMSCs was unveiled when a group recently discovered that transplanted BMSCs in ischemic rat hearts led to calcifications/ossifications [58]. This latter group stresses the importance of long-term studies, which will help determine the provenance, multipotency, and long-term fate *in vivo*. However, the same group discovered that use of cytokines derived from the BMSCs did not lead to these pathological malformations. Therefore, more data describing the long-term effects of administered MSCs and their factors are needed to be established. In addition, since BMSCs can be obtained directly from adult animal models, there are no pressing ethical issues limiting their usage in clinical trials. 

Although there are many benefits attributed to MSCs, it is important to know that the unique properties of MSCs do not guarantee that they will accomplish desired therapeutic outcomes or that they will evade rejection. This is the reason for the various conflicting results with regards to the mechanisms and outcomes of MSCs found in the literature. Hence, current research is highly focused on developing an efficient delivery system that can successfully transplant a sufficient number of stem cells at the desired site with higher retention and minimal biological loss or immune response. Some of our more recent work has shown that retention can be increased by four folds in the heart by using biocompatible polymeric alginate microcapsules as a delivery system, which can, in turn, help in functional recovery of acutely infarcted heart [59–61].

## 3. Exploiting the Cell Fusion and Differentiation Properties of BMSCs: Potentials and Limitations

Since heart disease is reaching pandemic proportions in developed and developing countries and the heart is an organ of limited regenerative capacity, much research using stem cell therapy is focused on the regeneration of the myocardium. Therefore, studies focusing on stem cell differentiation and cell fusion are often looking at the heart. In earlier studies concerning cardiac regeneration, successful transplantation of neonatal and fetal cardiomyocytes into rats with acute myocardial infarction was observed [62]. The results showed improved left ventricular function attributed to the formation of stable intracardiac grafts [63–67]. The implanted cells also retained their contractile phenotype and expressed necessary elements for intercellular electrical communication. 

Oric et al. demonstrated that bone marrow could be a source of extracardiac source of progenitor cells with the ability to differentiate into cardiomyocytes and restore cardiac function [9, 68, 69]. Since then, cardiogenic capabilities have been confirmed in hematapoietic stem cells, MSCs, circulating endothelial progenitor cells, and resident cardiac progenitor cells [11, 70–74]. Moreover, when the BMSC are transplanted into ischemic heart, they express the cardiac specific markers troponin I and cardiac myosin, allowing researchers to easily identify the transformation into functional cardiomyocytes and shows that histological difference is indeed occurring [71, 75, 76].

**Figure 1:**
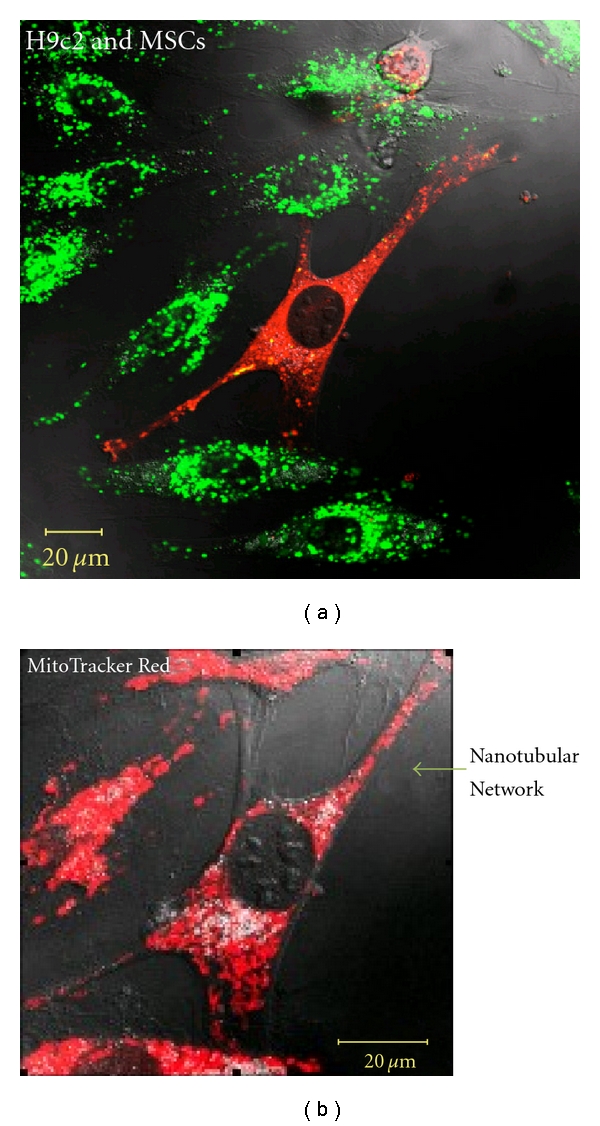
Formation of intercellular connections after oxygen glucose deprivation. (a) Nanotubular network formation was observed among DiO-labeled cardiomyoblasts (green) and DiD-labeled MSCs (red) after 24 hours of coculture. (b) MitoTracker staining (red) revealed active mitochondria in the nanotubular networks [5].

**Figure 2:**
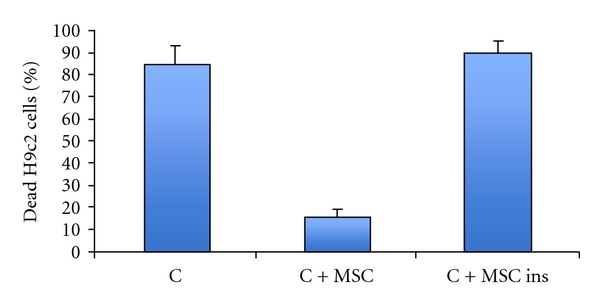
Cocultivation of cardiomyoblast (H9c2) cells with MSCs decreased cell death in oxygen glucose-deprived environment. (C): ratio of dead H9c2 cells after oxygen glucose deprivation. (C + MSC): ratio of dead H9c2 cells after co-cultivation with MSCs and under oxygen glucose deprivation (85 ± 8.6 versus 16 ± 3.5, *n* = 5). (C + MSC ins): ratio of dead H9c2 cells when MSCs were added with cell culture inserts (90 ± 5.5, *n* = 5). Data represent mean ± Standard deviation. *P < .05 C + MSC versus C and C + MSC versus C + MSC ins [5].

Cell Fusion of transplanted stem cells with resident cardiomyocytes has been proven as a feasible mechanism for differentiation. Fusion of bone marrow-derived cells with purkinje neurons, hepatocytes and cardiomyocytes was reported for the first time by Alvarez-Dolado et al. [77]. During cell, fusion the cells connect and exchange vital cell components [4, 78]. Cselenyák and others performed an elaborate *in vitro* experiment suggesting the presence of cellular nanotubes which increased the viability of ischemic cardiomyoblasts in the presence of normal MSCs [5]. It reveals that the viability of H9c2 rat cardiomyoblast cell line depends on the nanotubular connection with rat MSCs because when the nanotubes were blocked with cell culture inserts, the H9c2 viability decreased significantly.  Figure 1 shows cardiomyocytes and human mesenchymal cells communicating through small diameter nanotubes, which allow for the migration of organelles such as the mitochondria from MSCs to cardiomyocytes. The graph in Figure 2 illustrates the antiapoptotic effect of MSCs on stressed cardiomyocytes and how the nanotubular networks are crucial to their effects.

In a separate experiment, Noiseux et al. demonstrated, using a model of acute MI in transgenic mice, that transplanted MSCs could fuse with recipient cardiomyocytes in the infarcted area [79]. MSCs from wild-type C57BL/6 mice were retrovirally transduced to express green fluorescent protein (GFP) (reporter gene) and Cre recombinase. These MSC were transplanted into infarcted heart of histocompatible R26R mice. In these transgenic mice, a loxP-flanked stop sequence was present 5′ of the LacZ expression cassette to prevent transcriptional read through until selective excision by Cre-mediated recombination from implanted MSC. Thus, the LacZ gene was expressed exclusively after a donor MSC expressing Cre fused with a recipient cell from the R26R mice. Consequently, X-gal staining is used to detect cellular fusion events [77, 80]. This useful method, based on Cre/lox recombination that conditionally turns on the LacZ gene only in the fused cells, allows experimenters to rapidly observe tissue sections covering a large area of the infarcted and MSC-injected heart. The cells that were transplanted were found within the infarcted myocardium (detection of MSC by GFP immunoreactivity); early massive engraftment was observed at 3 days, but the number of implanted MSC decreased significantly over time, and by day 28 post-MI very few cells remained. As early as 3 days following MSC injection into ischemic heart, they observed cellular fusion with individual blue cells having typical cardiomyocytes morphology. Interestingly, the majority of the fusion events with LacZ^+^ cells were detected within the infarct border zone, in areas with viable cardiomyocytes. Although cell fusion was shown to occur by the appearance of blue cells (fusion of MSC with resident cardiomyocytes) and by the improved cardiac function, they concluded that fusion was not responsible because it happened so infrequently. Therefore, they attributed enhanced cardiac function to paracrine effects [79].

Despite hopeful results with experiments looking at fusion and differentiation, the mechanisms by which BMSCs work to help damaged tissue remains unclear even in seemingly successful studies. Furthermore, inconsistent results when researching stem cell regenerative capacity have been linked to improper labeling of donor cells and inability to consistently track them *in vivo,* making it very difficult to distinguish them from background tissue leading to misinterpretation [81]. For example, the utilization of the GFP reporter gene is attractive because it is compatible with a variety of imaging techniques; however dead and dying cardiomyocytes have an autofluorescent spectrum that partially overlaps with that of GFP. Therefore, after injury, autofluorescence increases due to accumulated lipofuscin, blood-derived pigments, and other intrinsic fluors such as flavins and reduced nicotinamide adenine dinucleotide (NADH) [82]. Evidence for regeneration includes colocalization of GFP fluorescence from donor cells, with immunostaining for cardiomyocyte markers, including sarcomeric actin cells. One major limitation of cell therapy is the low therapeutic efficacy of transdifferentiated cells in the site of injury. The low rate of sustained transplanted engrafted stem cells has been attributed to cell stress, hostile environment due to inflammation and hypoxia, insufficient blood supply, and elaboration of inflammatory cytokines resulting from ischemia and cell death [83–86]. In addition, overdiploid DNA fusion events between embryonic stem cells and BMSCs can result in cell fusion with 2 nuclei (2*n* + 2*n*), or alternatively nuclear fusion will result in cells with larger nuclei 4*n* chromosomes going up to hexaploid (6*n*) DNA content. This leads to hybrid genotypes with cells failing to express proper green fluorescent phenotypes adding to the confusion of tracking differentiated cells [87].

## 4. Exploiting the Paracrine Factors Released from BMSCs

Since research has shown that stem cell differentiation and fusion alone cannot account for enhanced cardiac function, evidence for a nonmyogenic pathway of cardiac repair is becoming increasingly explored. The debate surrounding fusion and differentiation is of little importance now because the number of reported cardiomyocytes derived from exogenous stem cells is too low to account for the impressive enhancement of physiological function [62, 88]. Thus, it has been proposed that stem cell transplantation possesses therapeutic effect because of the endogenous repair mechanisms secreted by the BMSCs that account for functional benefits of cell therapy in cases such as cardiac repair. 

MSCs such as BMSCs are known to secrete many biological factors, and under strenuous hypoxic and serum starvation, the expression of several of these factors is upregulated. This has prompted the isolation of MSC conditioned medium (MSC-CM) to be concentrated and used therapeutically. Kinnaird et al. reported the presence of cytokines as: VEGF, FGF-2, IL-6, PIGF, and MCP-1 and that intramyocardial injection of MSC-CM improved collateral blood flow recovery and limb function and reduced muscle atrophy. Because all these cytokines are known to promote angiogenesis and vascularization, the conclusion was that it was not direct cell incorporation but rather paracrine signaling that may be responsible for the effects of bone marrow cell therapy in ischemic injury.

**Figure 3:**
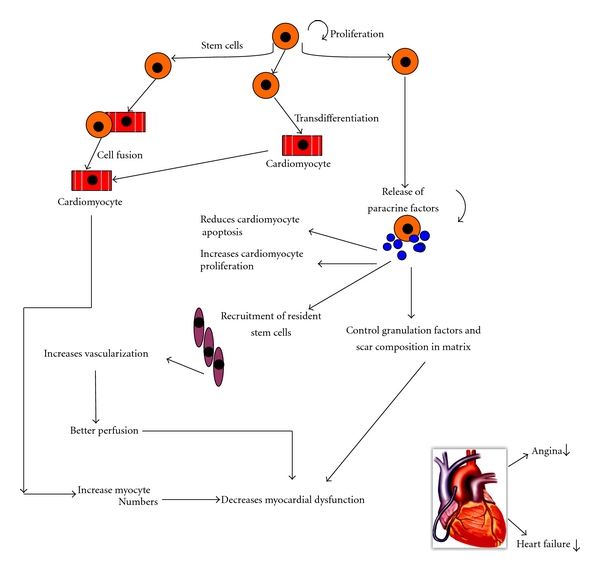
Schematic representation of the hypotheses presenting therapeutic effects of stem cell transplantation for myocardial regeneration. Cell transplantation can improve tissue perfusion and contractile performance by promoting formation of blood vessels and myocyte formation/protection. Central to the beneficial effects of cellular therapy is the paracrine and indirect effects of stem cells with the production and release of cytokines and growth factors. Depending on the stem cell type and local milieu, the relative contribution of cell incorporation (transdifferentiation and/or fusion) versus paracrine effects may vary [27].

It was previously reported that stem cell transplantation into the ischemic myocardium improved cardiac function recovery and repair as early as 72 hours later, which is too early to be explained solely by MSC differentiation [55, 56, 79]. To see how the MSCs confer protection of the ischemic myocardium through paracrine mediators and indirect effects, it was demonstrated that MSC-CM reduced hypoxia-induced apoptosis and triggered spontaneous contraction of isolated hypoxic adult rat cardiomyocytes *in vitro*, suggesting the presence of antiapoptotic and inotropic effects [55]. When injected directly into infarcted rat hearts, the MSC-CM limited infarct size as early as 72 hours and improved ventricular function at levels comparable to those observed following MSC transplantation. From this data, it is clear that the therapeutic effects observed after intramyocardial injection of MSC in the infarcted hearts are, at least, in part attributable to the paracrine effects on the ischemic myocardium. Paracrine factors can also play an important role in influencing the adjacent cells and exert their actions via diverse mechanisms. Furthermore, it is likely that the paracrine mediators are expressed in a specific temporal and spatial manner that can enhance cell survival and activate endogenous mechanisms of endogenous repair and regeneration [11]. Figure 3 provides a simplistic overview of the observed therapeutic potentials that arise from BMSC cardiovascular therapy and illustrates the direct and indirect influences of paracrine factors on overall cardiac functionality. Furthermore, Table 1 gives a brief overview of specific identified factors secreted by human MSCs detected by protein array [52].

### 4.1. Paracrine-Mediated Neovascularization

Blood vessel formation after birth proceeds primarily through 2 mechanisms: angiogenesis and arteriogenesis. Angiogenesis consists of several distinct processes which include sprouting and proliferation of pre-existing capillaries to form new networks. This process is tightly regulated by hypoxia through a number of proangiogenic factors, including VEGF, FGFs, P1GF, and hepatocyte growth factor (HGF) [89–94]. The rapid proliferation of pre-existing collateral arteries characterized by arteriogenesis involves remodeling small arterioles into larger vessels and is triggered in part by an increase in stress in arterioles that run parallel to the occluded artery. Recruitment of monocytes that differentiate into macrophages and produce abundant angiogenic growth factors such as VEGF, NO, MCP-1, and other cytokines, is also essential and ultimately leads to endothelial and smooth muscle proliferation, migration, vessel remodeling, and extracellular matrix synthesis [44, 94]. Arteriogenesis is the most efficient adaptive mechanism for the survival of ischemic organs because of its unique capability to conduct large blood flow. Angiogenesis and arteriogenesis are complex processes sharing common mechanisms of action, growth factors, and cytokine dependency. Furthermore, these cytokines act not only in a coordinated time and concentration-dependent manner, but one cytokine may inhibit or stimulate the effect of another. The complexity of the process of collateral formation has led to the suggestion that multifactorial strategies will be necessary to modulate therapeutic paracrine effects including vessel formation. 

Under normal circumstances, myocardial tissue dies and forms scar tissue because the capillary network does not meet the demands of the hypertrophied cells. The inevitable lack of oxygen and nutrients leads to apoptosis and necrosis. Moreover, inducing angiogenesis requires the coordinated interactions between monocytes/macrophages, endothelial cells, smooth muscle cells, and pericytes [92, 94–99]. Human bone marrow-derived multipotent stem cells allow for angiogenesis by expressing various angiogenic cytokines such as VEGF, Ang-1, Ang-2, bFGf, HGF, and PDGF-B. More importantly, HGF, VEGF, bFGF, and IGF are known survival factors that are expressed by cardiomyocytes [96, 97]. These paracrine factors are believed to influence adjacent cells via mechanisms including myocardial protection, neovascularization, and most the activation of resident cardiac stem cells and/or stimulation of endogenous cardiomyocyte replication. 

MSCs express genes encoding a broad spectrum of cytokines with angiogenic properties of some of which may be referred to in Table 1 such as VEGF, HGF, FGF, MCP-1, PGF, IL-1 and IL-6, SDF-1, and MMP-9 [37, 44, 47]. All of these cytokines can be isolated from the media of cultured cells and have all shown to have positive effects on experimental blood flow recovery [37, 91]. In addition, MSC-CM promotes *in vitro* proliferation and migration of endothelial cells and vascular smooth muscle cells and enhances *in vivo *collateral blood flow recovery when injected into ischemic hindlimb. Another possibility for improved function is that neovascularization by the stem cells is leading to enhanced blood supply in the peri-infarct region, thereby promoting salvage of stunned, hibernating, or otherwise susceptible cardiomyocytes [62].

### 4.2. Paracrine-Mediated Cell Homing/Targeting

Homing signals are extremely important for the efficacy of cell therapy because it is via these paracrine effects that the precise localization of transplanted cells is possible and can be improved. In the case of cardiac repair, stem cell therapy can be administered via intravenous, intracoronary, transmyocardial, catheter-based transendocardial with or without electromechanical voltage mapping, and finally transvenous injection into coronary veins [100–104]. The goal of any cell delivery strategy is to transplant enough cells into the area of interest to achieve maximum retention in that area. Moreover, the local environment determines the success of cell delivery since it is the milieu that will influence short-term cell survival, cell properties in regard to adhesion, transmigration through vasculature, and tissue invasion. Therefore, the targeted administration of cells is preferred [105]. Cell homing, transmigration, adhesion, and tissue invasion are the result of many complex steps. For example, stromal-derived factor-1 (SDF-1) is expressed in the ischemic myocardium and plays an important role in endogenous stem cell migration, adhesion, homing, and recruitment from bone marrow. SDF-1 is an important chemoattractant for progenitor cells and thus plays a crucial role in directing cells into areas of infarcted myocardium [75]. Tang and colleagues administered SDF-1 expressing plasmid into ischemic border zones of murine models injected with BMCs (Lin^−^c-kit^+^) [106]. A significant amount of the labeled stem cells were found in the area where the plasmid was injected. Thus, administering homologous cytokines is one way to encourage the proliferation of injected stem cells.

Of all techniques, the intracoronary infusion has been deemed best for treatment of acute myocardial infarction. However, strategies to augment cell function via better localization/homing is crucial if there is to be any therapeutic benefit using BMSCs for targeted regenerative therapy. Additional strategies for better homing of mesenchymal cells include inducing hypoxia for the upregulation of CXCR-4 protein receptor expression at the BMSC cell surface. CXCR-4 are chemokine protein receptors expressed in dying cells, which can be complexed with SDF-1 protein ligands [107, 108]. The CXCR-4/SDF-1 complex allows for stem cell migration via chemotaxis where expression of the CXCR-4 receptor and the presence of SDF-1 receptor are required to regulate and make cell migration possible. It has been found that CXCR-4 expression can be induced by exposure to a pool of cytokines such as SCF, IL-6, Flt- ligand, HGF, and IL-3. Furthermore, increasing affinity of the stem cells can also be done by increasing the concentration of the chemo-attractant SDF-1. Therefore, a depot of angiogenic cytokines and an increase in SDF-1 ligand can possibly be localized in an area, allowing for efficient stem cell homing [109]. 

Orlic et al. reported that injection of BMCs (Lin^−^c-kit^+^) was able to give rise to new myocardium and improve left ventricle end diastolic pressure by 30% to 40% in mouse hearts with infarcts located in the free wall of the left ventricle [1, 110]. In a separate but similar experiment, injections of the BMSC cytokines stem cell factor (SCF) and granulocyte-colony stimulating factor (G-CSF) increased numbers of circulating HSCs to a level 250-fold greater than untreated myocardium. However, it is not clear whether tissue repair is a result of the homing of BMC to the lesion or if BMC, once mobilized, cells spreads through the organism and only those exposed to damaged tissue are triggered to proliferate and differentiate at a faster rate than the others [9]. Nonetheless, the former hypothesis is supported because of the rapid induction of SCF in many tissues including the myocardium. Therefore, SCF could be responsible for migration, accumulation, and multiplication of primitive BMCs in the infarcted zone where they acquire the appropriate phenotype.

### 4.3. Paracrine-Mediated Anti-Inflammatory Effects

MSCs are known for having potent anti-inflammatory activities, which are present regardless of their tissue of origin. They are said to suppress inflammation through secretion of anti-inflammatory cytokines such as IL-10, TGF-beta, LIF, soluble HLA-G, and IL-1 receptor antagonists [111]. The successful effects of MSC have been demonstrated *in vivo* and have treated animal models in cases of immune mediate/inflammatory pathologies such as MS, colitis, graft versus host, rheumoid arthritis, and ischemic injury [112]. Most recently, Chen et al. have shown that MSCs implantation after myocardial infarction in a rat model caused a significant upregulation of anti-inflammatory versus the proinflammatory cytokines compared with the control group without cell therapy [113]. It is therefore conceivable that MSC factors can also work by downregulating the genes, which promote inflammation and therefore are useful against heart failure by suppressing ongoing self-perpetuating inflammatory cascades. 

As it has been aforementioned, knowing the microenvironment prior to cell transplantation or conditioned media transplantation is crucial to avoid any immune-mediated rejection. That being said, inclusion of specific cytokine receptor antagonists or inhibitors can prevent the immune-mediated responses of the host microenvironment on transplanted cells. Since IL-1*α*/*β*, TNF-*α*, and SDF-1-*α* are ubiquitous in the cell microenvironment, we must review their mechanisms. First of all, these are pro-inflammatory mediators, which are primarily synthesized by macrophages, monocytes, and dendritic cells, and are responsible for immune defense against infection in most tissues [114]. Greco and Rameshwar have shown that MSCs express IL-1RI, which is the principal receptor for IL-1*α* and IL-1*β* [32]. The researchers found that membrane expression of the receptor was maintained throughout transdifferentiation of MSCs into neurons. Moreover, IL-1*α* was found to alter behavior of undifferentiated MSCs. Stimulation of MSCs with IL-1*α* caused production of substance P neurotransmitter by undifferentiated and differentiated cells. Excessive production of substance P neurotransmitter leads to exacerbated immune responses and untoward effect on MSC differentiation. On the other hand, delivering MSCs together with inflammatory cytokine antagonist/inhibitor may suppress immunoreactivity allowing for the expected therapeutic result [115]. Therefore, knowing the correct inhibitors will assure optimal functioning of transplanted cells or media. 

As demonstrated *in vitro* and *in vivo*, it is now known that BMSCs can be immunosuppressive and escape cytotoxic lymphocytes [116, 117]. One mechanism by which immunosuppressive effects are mediated is by Fas-mediated apoptosis. Cells expressing FasL specifically protect against T-cell-mediated cytotoxicity where T cells expressing Fas are sensitive to Fas-mediated apoptosis. One study hypothesized that this immunosuppressive action is mediated by FasL-induced killing of activated lymphocytes, thereby, preventing lymphocyte attack. It has been suggested that cell engraftment can be improved by the pretreatment of cells with anti-apoptotic or “cytoprotective” genes; some of which can be found in stem cell media (Akt, Bcl2 GSK, IlK, telomerase, and eNOS) [118].

### 4.4. Paracrine-Mediated Endogenous Cell Stimulation

Activating specific cells through paracrine/endogenous effects of growth factors released by BMSC is showing promise. The kidney, similar to the heart, has a limited regenerative capacity. However, recent studies have suggested the presence of stem cell renal niches, that is, renal papillae in animals and in the urinary pole of Bowman's capsule in humans. The importance of these CD24+ and CD133+ stem cells during kidney damage has not yet been elucidated, and it has been found that MSCs contribute to renal repair [119]. Similarly to cardiac treatments, when treating the kidney with transplanted MSCs, several studies documented renal recovery without differentiation of MSCs into epithelial cells. Therefore, it was also suggested that cell migration facilitated regeneration because of endocrine/paracrine effects and that it was the kidney stem cells that were re-generating the tubular epithelium [120–125]. Furthermore, the body also possesses local source of cardiac stem cells, which can differentiate into myocytes and possess growth factor-receptor systems allowing for the regeneration of infarcted myocardium. It is known that HSCs and MSCs can stimulate activation of these cardiac resident stem cells because they can produce HGF and IGF-1 which are not only anti-apoptotic but can activate endogenous stem cells [126]. Interestingly, these factors are upregulated in response to inflammatory mediators such as TNF-alpha. Therefore, when responding to inflammatory stimuli, MSCs and HSCs can, in addition, potentially recruit endogenous stem cells [109].

## 5. Recent Applications of Stem Cell-Conditioned Media in the Biomedical Fields

### 5.1. Cardiac Regeneration

Although this is a novel topic, some studies have been done using BMSC-derived conditioned media (BMSC-CM). Their preliminary results suggested that much more efforts should go into further researching the benefits of conditioned media to better understand the role of individual factors that may optimize their effects. Interest in this topic started when researchers reported functional improvement of tissue before the possibility of attributing it to donor cells. Gnecchi and colleagues found significant functional improvement in the myocardium occurring in less than 72 hours after intramyocardial injection of Akt-modified MSCs in a rat model [56]. The same group later observed that cell fusion events were occurring and were associated with better cardiac performance. However, the cell fusion and differentiation events were so uncommon that they attributed the enhanced cardiac function to paracrine factors expressed by MSCs [48, 79]. Nguyen et al. are the first to research the effects of MSC-CM on the myocardium [52]. After subjecting swine to acute MI by balloon occlusion of the left anterior descending coronary artery, the effects of intracoronary injections of either concentrated MSC-derived growth factors or control medium were observed. MSC-derived factors significantly reduced serum cardiac troponin T (cTnT) that has been validated as a biomarker that increases with the severity of injury. The treatment group also showed significant echocardiographic functional improvement. Staining analysis also revealed a reduction of the fibrotic area in the infarct zone and increment of preserved area in the infarcted myocardium. This was associated with less apoptosis of cardiomyocytes. The roles of angiogenic (vascular endothelial growth factor, endothelin, and epiregulin), anti-apoptotic (Galectin-3, Smad-5, sRFP-1, and sRFP-4) and anti-remodeling factors have all been detected by protein array in the control group. 

Recent research has also shown that there is an optimal time for conditioned media delivery since the paracrine effects are known to be time dependent. It has been demonstrated that the prohypertrophic effects on myocytes and proliferation of fibroblasts in rat myocardium were significant using media collected after 6 and 9 hours of secretion. On the other hand, medium collected at 3 and 24 hours after secretion resulted in no noticeable prohypertrophic effects. Consequently, the presence of FGF-2, IL-1, IL-6, TGF-*β* and TNF-*α* factors during these optimal times suggests that they all take part in hypertrophy and proliferation of the cardiomyocytes [127].

### 5.2. Renal Regeneration

Similarly to stem cell therapy in myocardial regeneration, studies in the kidney provided no evidence that marrow stromal cells were incorporating into the native renal parenchyma in significant numbers or directly contributed to tubular cell repopulation [120]. Bi and colleagues studied whether BMSC-CM injections had any beneficial effects on the renal system. Using in vitro mouse proximal tube cells (MPT) cultured (24 hours) with cisplatin with and without BMSC-CM, they showed that sustained exposure to toxic cisplatin resulted in detachment and death of many MPTs. The presence of BMSC-CM, however, significantly inhibited cell detachment and inhibited cell death after 48 h. The same study investigated the mechanisms of renal repair after BMSCs injection. They showed no evidence of renal cell regeneration but rather the endocrine effects conferred the greatest therapeutic benefit for the cells [125].

### 5.3. Neurotrophic Actions of BMSC-CM

Bone marrow stromal cells also showed great potential with regards to neurological regenerative therapy. BMSCs have shown to contribute to functional recovery and tissue regeneration in rat models with spinal cord injury, cerebral ischemia, traumatic brain injury, and constructing tissue-engineered nerve grafts [128–130]. Researchers have investigated the paracrine factors released by BMSC-CM using rat dorsal root ganglion cells. They cocultured BMSC-CM with explants or neurons, and cell proliferation assays showed that neurite outgrowth and neuronal cell survival were enhanced. Because they used transwell inserts and BMSC-CM, the cell behaviors of explants and neurons were induced by soluble factors released by BMSCs into the culture medium. The factors contributing to immune cell migration to injury site, remodeling, and cellular action which all contribute to repairing injured nerves can be attributed to the factors exerting paracrine factors such as 43 secretory proteins, adhesion molecules, cytokines, and growth factors. Furthermore proteomic analysis and ELISA further found that BMSC-derived soluble factors were involved in activation of PI3-K/Akt and MAPK signaling pathways in DRG neurons. The activation of PI3-K/Akt and MAPK/Erk1/2 pathways is crucial for survival signaling. Therefore, these results suggested a new pathway by which cell survival might be attributed to endocrine paracrine mechanisms [131].

## 6. Future Directions

### 6.1. Advanced Cell Isolation Methods for Enhancement of Paracrine Activities

Optimizing and maximizing the amount of secreted stem cell paracrine factors is important to assure its efficient transition in therapeutic and clinical applications. Preparation and isolation of MSC has traditionally involved plastic adherence isolation. Another method for cell isolation is by immunoselection using mesenchymal precursor cells (MPC) at higher purity. MSC involving plastic adherence (PA) isolation has been shown to be less effective than MPCs immunoselected for stromal precursor antigen-1 (STRO-1) [132, 133]. Thus STRO-1-MPC displayed greater clonogenecity, proliferative capacity, multilineage differentiation potential, and mRNA expression of mesenchymal stem cell related transcripts. As a result, STRO-1-MPC exhibited higher gene and protein expression and thus greater paracrine activity than PA-MSC *in vivo*. More importantly, enrichment for STRO-1 increases the expression of cardiovascular-relevant cytokines and enhanced trophic activity. STRO-1-MPC, for example, it exhibited higher expression of CXCL12 and HGF, which are crucial in endothelial tube formation and cardiac cell proliferation. Therefore, immunoselection has great potential in the field of regenerative therapy.

### 6.2. Nanobiotechnological Applications

Enhancing effectiveness of paracrine activity has also been attempted using heparin-binding amphiphile nanoparticles (HBPA) [134]. Hypoxic-conditioned medium was derived from MSCs which led to significantly better haemodynamic performance at 30 days after infarct in a mouse ischaemia-reperfusion model of acute myocardial infarction. Interestingly, enough MSC-CM-HBPA animals did not vary significantly from healthy animals with respect to LV contractility; thus, HBPA nanofibre networks resulted in significantly greater myocardial functional performance than with HBPA alone or with conditioned medium alone. The mechanism by which the synergistic effects of CM and HBPA functioned was likely due to the heparin-containing nanofibre gel concentrating heparin-binding factors, stabilizing factor-receptor complexes, prolonging factor activity by suppressive proteolysis, and increasing retention through the introduction of molecular aggregates rather than isolated proteins. The study also showed that loading HBPA nanofibre networks with nanogram quantities of growth factors VEGF and bFGF was enough to recreate the enhanced cardiac function. However the mechanism by which cardiac function was improved was not well described especially because they did not notice any changes relating to angiogenic factors. Therefore, they postulated that the HBPA nanofibre in synergy with the conditioned media augmented function via trophic effects which has been shown to augment function by modulation of cardiac metabolism. In other words, this new idea proposes that paracrine factors might help erase ischemic memory and help the heart utilize the cardiometabolic reserves [135].

## 7. Conclusion

So far, bone marrow-derived stem cells and their paracrine factors have shown all the necessary attributes for tissue regeneration, namely, homing, immunosuppression, differentiation, angiogenesis, stimulation of endogenous cells, and possible regulation of specific metabolic pathways, only to name a few. Thus, research into paracrine factors and mechanisms has shown that stem cell therapy is much more complicated and greatly enhances the potential and variety of therapeutic applications. Mediating angiogenic factors, cell proliferation, and all of the above-mentioned characteristics are crucial to stem cell therapy and provides many new approaches for therapy. Therefore, injecting MSCs into tissue and observing the extent of differentiation are to be considered superfluous unless, of course, paracrine effects are being considered. Results from experiments using only conditioned medium showed signs of remarkable promise and revealed the same regenerative capacities as studies using cell injection. However the hallmarks of transdifferentiation and cell fusion are not to be disregarded because paracrine activities will most likely make these processes more efficient. Therefore, research into using conditioned media from stem cells is crucial because it will give us a greater understanding into the most relevant and important cytokines, their optimization, and their mechanisms of action to further maximize the application and uses of regenerative stem cell therapy. 

## Figures and Tables

**Table 1:** Detection of secreted factors from human mesenchymal cells under normoxic and hypoxic conditions. Relative concentration between normoxic and hypoxic conditions are expressed from 1–5 with 1 being the lowest concentration and 5 being the highest [52].

Secreted factors	Normoxia	Hypoxia	Biological function
*Activin A*	3	2	Cell proliferation, differentiation, apoptosis, and immune response
*Epiregulin*	3	3	Remodeling
*Endothelin*	4	4	Cytoprotection, cell proliferation
*Glypican-3*	3	3	Cell proliferation
*IGFBP-7*	4	5	Cytoprotection, cell migration, and contractility
*IL-15 alpha*	2	1	Immune response
*LRP-1*	2	1	Cell migration
*LRP-6*	4	1	Cell migration
*Osteoprotegrin*	4	5	Bone development
*sFRP-4*	3	4	Development, apoptosis
*Smad4*	3	2	Vessel maturation, cell proliferation
*Smad7*	2	2	Vessel maturation, cell proliferation
*Thrombospondin-1*	3	5	Cell migration, apoptosis
*TIMP-1*	4	5	Cell migration, remodeling
*TIMP-2*	4	5	Cell migration, remodeling
*VEGF*	3	4	Cytoprotection, proliferation, migration, and angiogenesis
